# Supplementary material for: Complexome profiling of the Chlamydomonas psb28 mutant reveals TEF5 as an early PSII assembly factor
Source: Plant Cell. 2025 Mar 17;37(6):koaf055. doi: 10.1093/plcell/koaf055 (PMC12164590; doi:10.1093/plcell/koaf055)
Supplement: koaf055_Supplementary_Data [file koaf055_supplementary_data.zip › psb28_interactive.zip!/plots/intensity/qualityPlots/peptides/CYC1_Cre15.g638500.t1.2.html]

### Cre15.g638500.t1.2

Trivial name: CYC1  
SeqCoverage [median]: 29.60  
Mass: 33.83 kDa  
  
 

| Sequence | MissedCleavage | Mass [Da] | wtIntensity | muIntensity | Score |
| --- | --- | --- | --- | --- | --- |
| ALAAETEVEDGPNDEGEMFTR | 0 | 2279.99 | 51364.33 | 59474.34 | 222.79 |
| DPPAGISIR | 0 | 924.50 | 872.99 | 0.00 | 59.35 |
| EGLYYNPYFPGGAIAMPK | 0 | 1986.95 | 27306.16 | 5850.66 | 144.57 |
| LFDAFPSPYANEQAAR | 0 | 1795.85 | 62671.28 | 19723.65 | 190.66 |
| MLVDGGVEYEDGTPASASQQAK | 0 | 2252.03 | 41435.08 | 39447.13 | 183.39 |
|  |  |  | 183649.8 | 124495.8 |  |
